# Electroencephalography-based neural indicators of texture preference for cosmetic formulations

**DOI:** 10.3389/fnins.2025.1620806

**Published:** 2025-10-29

**Authors:** Hye-Ran Cheon, Gusang Kwon, Youngkyung Kim, Hyunjung Kim, Hae Kwang Lee, Jin Hee Shin, Joomi Yu, Han-Jeong Hwang

**Affiliations:** ^1^Department of Electronics and Information Engineering, Korea University, Sejong, Republic of Korea; ^2^Interdisciplinary Graduate Program for Artificial Intelligence Smart Convergence Technology, Korea University, Sejong, Republic of Korea; ^3^AmorePacific, Seoul, Republic of Korea; ^4^Department of Dermatology, Chungnam National University School of Medicine, Chungnam National University Sejong Hospital, Sejong, Republic of Korea; ^5^P&K Skin Research Center, Seoul, Republic of Korea

**Keywords:** formulation, preference, electroencephalography (EEG), cosmetic, correlation

## Abstract

This study investigated the correlation between subjective preferences for different cosmetic formulations and brain activity measured using electroencephalography (EEG). EEG data were collected from 29 participants when they applied three positive and one negative cosmetic formulation to the inside of their left forearms. According to the questionnaire results, the negative formulation showed significantly lower preference scores than the positive formulations. Additionally, significant EEG-preference correlations were consistently found in the delta and alpha bands within the sensorimotor areas closely related to tactile processing and its emotional regulation. In particular, stronger correlations were observed when only the two positive formulations with higher preferences were included in the analysis or when specific frequency bands showing significant results were combined together. These findings demonstrate the potential of predicting cosmetic preferences based on EEG data and highlight the crucial role of texture sensation in shaping user choice.

## Introduction

The market for cosmetic products has significantly expanded in recent years, giving consumers a broader range of options. However, this abundance has also made it more challenging for consumers to identify products that match their skin types and preferences. It is nearly impossible to try every available product, and given variability in individual skin conditions, relying solely on others’ reviews has inherent limitations. In response, several cosmetic companies have begun offering personalized products. For instance, Amorepacific, a leading cosmetic firm in South Korea, has developed solutions tailored to the skin tones of Asian women (Color correcting technology, 2025), and introduced 3D printing technology for customized facial masks (Tailored facial mask pack 3D printing system, 2025). Another company, Dr. JCOS, has created a big data-based system to analyze global skin characteristics for personalized recommendations (Dr.JCOS INC., 2025).

In the development of personalized cosmetic products, both external factors (e.g., skin type) and internal factors (e.g., emotions and preferences) play crucial roles. Conventional methods such as questionnaires or focus group interviews have been commonly used to assess consumer preferences (Frey and Fontana, 1991; Cavill and Bryden, 2003). While these approaches can gather a variety of information, respondents may withhold or inaccurately report their genuine feelings due to self-consciousness or uncertainty about their own preference. Moreover, because these methods rely on retrospective recall rather than real-time reactions, time-related distortions can occur.

To address these limitations, researchers have been exploring neurophysiological approaches, such as electroencephalography (EEG), functional magnetic resonance imaging (fMRI), and eye tracking, to measure consumer emotions and preferences in real time (Phan et al., 2002; Bos, 2006; Ares et al., 2014; Jenke et al., 2014). These approaches, now widely adopted within the framework of neuromarketing, enable the capture of subconscious responses that consumers themselves might not recognize, providing more objective and quantifiable data in the context of cosmetic product usage. Among these neurophysiological techniques, EEG is particularly notable for its high temporal resolution, which allows for the detection of rapid responses to specific stimuli. It is also more accessible than many other neurophysiological modalities due to its relatively lower cost and fewer spatial constraints. Owing to these methodological advantages, EEG has been extensively utilized across multiple neuromarketing contexts to investigate consumers’ implicit emotional and cognitive responses (Yadava et al., 2017; Khurana et al., 2021; Khondakar et al., 2024).

A recent review examined neurophysiological research on emotional responses to cosmetic products conducted before May 2024, identifying 33 relevant studies (Diwoux et al., 2024). Of these, only 12 utilized EEG, highlighting the limited number of EEG-based investigations in this field. After excluding studies focusing on specific cosmetic compounds or solely on classification, only four explicitly investigated emotional responses induced by cosmetics themselves. Two of them centered on fragrances; for instance, one study showed that inhaling a lavender-scented cleansing gel induced a positive mood, as evidenced by changes in frontal alpha asymmetry (Field et al., 2005). Another study found that inhaling certain fragrances affected alpha activity, indicating relaxation effects (Churchill and Behan, 2010).

Fragrance is undoubtedly an important factor in cosmetic product selection. However, texture, which varies according to different formulations, also plays a critical role. Despite this, relatively few studies have explored the neurophysiological mechanisms associated with formulation-specific textures, especially when compared to visual and olfactory stimuli, which have been more extensively studied in the field of neuromarketing (Golnar-Nik et al., 2019; Cuesta et al., 2020; Ouzir et al., 2024). For example, one of the studies mentioned in the review assessed both subjective preferences and EEG responses for various formulations, but it relied solely on EEG data to interpret emotional states (Gabriel et al., 2021). Another study explored the correlation between subjective valence and EEG-based valence, but because the product included scent and color, the findings did not isolate the effects of formulation alone (Wang et al., 2024). Therefore, there is limited quantitative evidence demonstrating whether EEG can reliably capture formulation-specific emotional responses. Furthermore, to our best knowledge, no study to date has established a clear neurophysiological interpretation by correlating subjective preferences with EEG signals exclusively in the context of cosmetic formulations.

In this study, therefore, we measured brain activity induced by applying different cosmetic formulations–explicitly excluding external factors such as scent and color–and investigated how these EEG measures correlate with subjective preferences collected via a standard questionnaire. To this end, EEG data were measured while thirty-two participants applied three positive formulations and one negative formulation, followed by a self-report survey on preference. We then compared subjective preference levels among the four formulations and performed correlation analyses between these preference levels and the corresponding EEG signals. Through this study, we aim to demonstrate the potential of neurophysiological measures as a valuable complement to traditional approaches in evaluating preferences related to cosmetic products.

## Methods

### Participants

Thirty-two healthy female participants aged between 25 and 45 years (mean age: 34.47 ± 6.10 years) participated in this study. Due to the relatively higher frequency and interest in the use of cosmetics products among women compared to men, only females were selected as participants. None of the participants had any acute or chronic physical diseases, including infectious skin disease, skin allergies, and neurological or psychiatric disorders. Subjects were recruited through the website of the P&K Skin Research Center, where the experiments were carried out. The local Institutional Review Board (IRB) approved the study protocol (2021-1CR-N38S). All participants were fully informed about the purpose and details of the study and voluntarily signed a consent form.

### Experimental procedures

Before the experiment, four locations on each participant’s left inner forearm were marked with a 50 mm diameter stamp, ranging from the wrist to the elbow. This ensured the proper application of four different cosmetic formulations: three positive formulations (P1, P2, P3) and one negative formulation (N). The positive formulations were characterized by moisture and a smooth texture, with similar hardness and pH values. In contrast, the negative formulation was oily, had poor spreadability, a relatively high hardness value, and lacked moisture. These characteristics were expected to induce positive and negative preferences, respectively. Among the four cosmetic formulations used in the experiment, three formulations (P1, P2, N) were based on commercially available products, while the remaining one (P3) was a prototype of the cream product. To eliminate possible olfactory and visual influences and focus on the tactile experience related to the texture of the cosmetic formulations themselves, all fragrances and colorants were completely removed. Table 1 provides details on the four formulations used in this study.

**Table 1:** Information regarding the four formulations used in the study.

Formulation	Product name	Brand	Hardness	pH
P1	Primera Oil-Free Gel Cream* (Alpine berry watery oil-free gel cream, 2025)	AmorePacific	16	6.80
P2	Hanyul Pure Artemisia Calming Water Cream (Pure artemisia calming water cream, 2025)	AmorePacific	16	6.00
P3	Ceramide Beta cream #14 **	AmorePacific R&I Center	16	7.01
N	Aquaphor Baby Healing Ointment (Aquaphor® baby healing ointment (14 oz.), 2025)	Beiersdorf	375	Unmeasurable (no moisture)

Hardness was measured using the Compac-100 II (Sun Scientific, Japan), and pH was measured using the S20-K SevenEasy™ (Mettler-Toledo, USA). Based on the hardness and pH values, P1, P2, P3 formulations were expected to induce positive preferences, while N formulation was expected to induce negative preferences. *indicates a product that is currently discontinued; ** refers to a prototype formulation that is not commercially available. The pH value of the N formulation was unmeasurable due to its thick, paste-like texture and lack of moisture content.

Each formulation was applied five times in a randomized order to prevent potential order effects. The experimenter used a pipette to apply a fixed amount (50 μL) of each formulation to the designated locations on the left forearm. Participants were instructed to rest for 5 s and then apply the formulation to their skin with their right hand for 30 s. To induce natural emotional responses, they were instructed to apply the formulations according to their usual habits, without restrictions on application methods (e.g., tapping, rubbing, or the speed of application). This design choice was intended to enhance ecological validity—that is, to ensure that the experimental conditions closely resembled real-life usage scenarios—by allowing participants to interact with the products in a natural and familiar manner, rather than imposing artificial constraints on their behavior (Subramanian et al., 2023).

**Figure 1:**
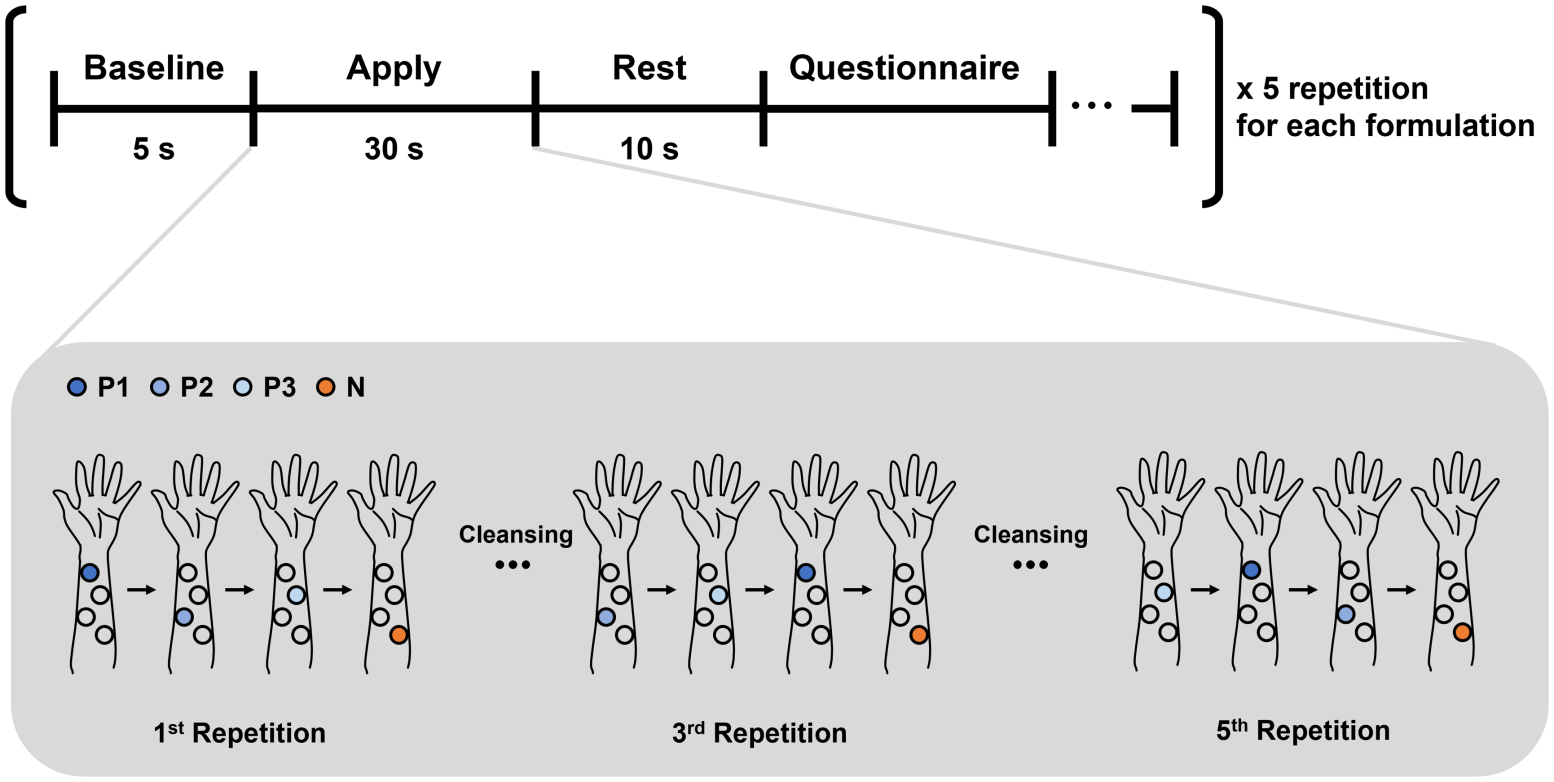
Schema of the experimental paradigm. After a 5-s baseline period, participants applied a cosmetic formulation for 30 s. Following a 10-s rest period, they evaluated their preference for each formulation using a questionnaire. Within each repetition, four formulations were applied in randomized order to pre-marked skin areas. After each application, participants wiped their fingers with a wet tissue to remove any residue before applying the next formulation. To minimize carry-over effects, the N formulation–because of its sticky, paste-like consistency that was more difficult to clean–was always applied last, ensuring that incomplete removal would not affect subsequent applications. After completing one repetition, all applied skin areas were also gently wiped with a wet tissue. Each formulation was tested five times.

The overall experimental procedure was conducted following the sequence illustrated in Figure 1. In each repetition, the four formulations were applied once in randomized order to the four marked areas. Because the N formulation had a thick, paste-like consistency that made it difficult to remove cleanly, it always applied last. After completing one full repetition (i.e., one application of each formulation), the skin was gently wiped with a wet tissue to minimize residual effects before the next repetition began. To further minimize cross-contamination, participants were instructed to wipe their fingers with wet tissues after each application, ensuring that residue from one formulation would not affect the next.

After each application, participants completed a preference questionnaire using PsychoPy (2025). The questionnaire used a 7-point Likert scale (1 = “non-preferred,” 7 = ‘highly preferred”). After the experiment, three participants were excluded from the analysis for the following reasons. For Sub 1 and Sub 6, the formulation order information and some experimental data were not recorded due to technical issues, respectively. Sub 10 was excluded due to inconsistent preference scores of the formulations. As a result, the questionnaire and EEG data from twenty-nine participants were included in the final analysis.

### EEG data acquisition and pre-processing

**Figure 2:**
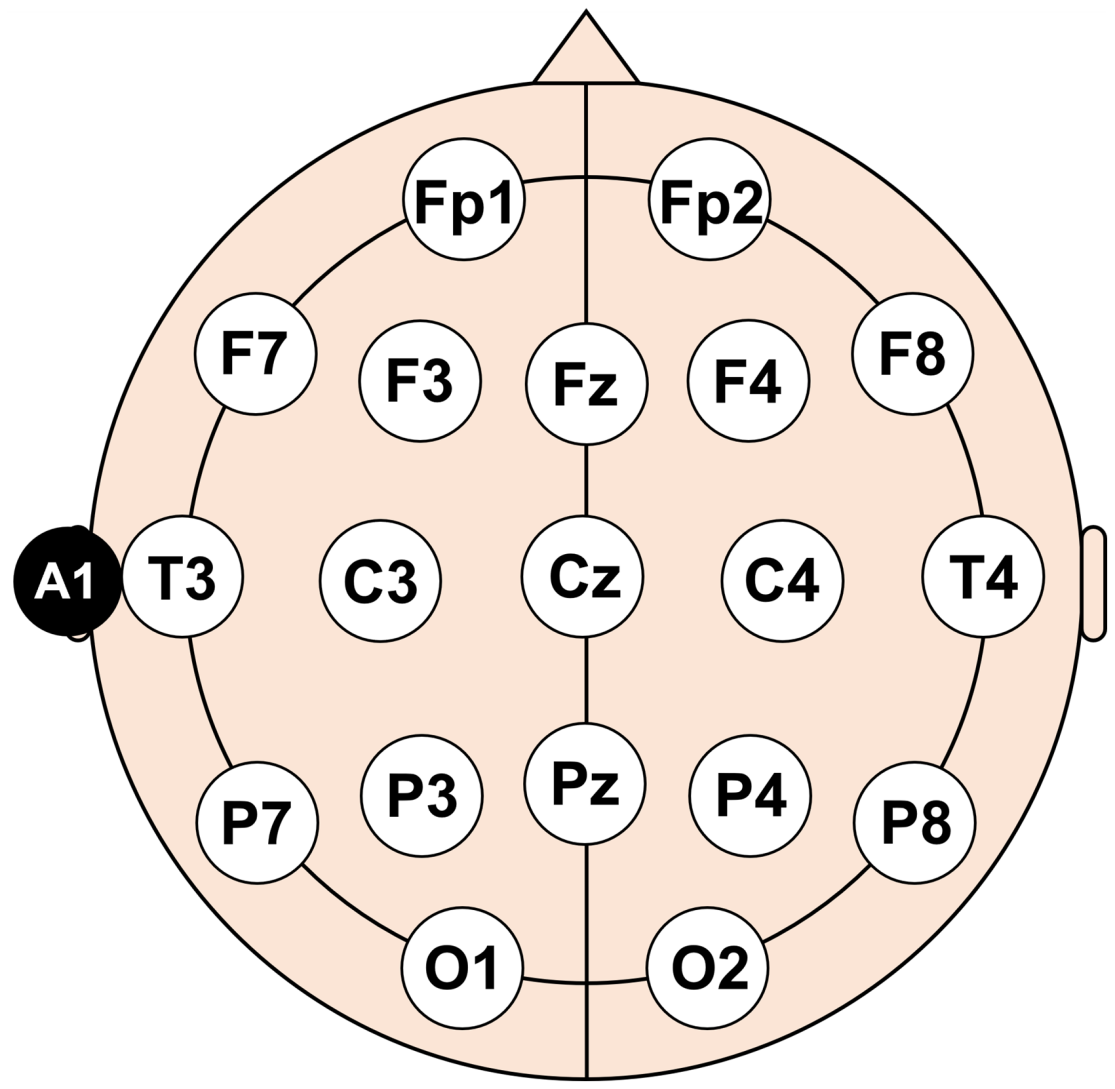
Electrode positions.

EEG data were acquired using a CGX Quick-20r system (Cognionics Inc., San Diego, CA, USA) equipped with nineteen dry electrodes (Fp1, Fp2, F7, F3, Fz, F4, F8, T3, C3, Cz, C4, T4, P7, P3, Pz, P4, P8, O1, O2), attached on the scalp according to the international 10–20 system (Dry EEG headset, 2025). The A1 electrode was placed on the left earlobe served as both ground and reference (Figure 2). Raw EEG data were re-referenced using common average reference (CAR) to minimize potential lateralized artifacts that could arise from unilateral referencing (e.g., A1). The data were then band-pass filtered between 0.5 and 55 Hz, and down-sampled to 200 Hz to reduce data volume. Considering the sensory adaptation effects, which typically result in a progressive reduction of sensory-related neural activity during prolonged stimulation (Bradley et al., 2016; Rideaux et al., 2023) the filtered EEG signals were epoched from −1 to 20 s relative to the onset of the 30-s application period. Independent component analysis (ICA) was then performed to remove physiological artifacts, such as electrooculography (EOG) and electromyography (EMG). The P3 electrode was excluded from further analysis due to severe physiological artifacts. For individual participants, any channel with severe noise was also excluded (Sub 27: P8 and O1; Sub 29: O1). Any remaining epochs with residual artifacts after the pre-processing steps were additionally removed from visual inspection.

### EEG analysis

We performed event-related spectral perturbation (ERSP) analysis to obtain event-related (de)synchronization (ERD/ERS) values. ERSP analysis was conducted on EEG data epoched from −1 to 20 s relative to stimulus onset to avoid potential adaptation effects that may occur during the latter part of the 30-s period. The −1 to 0 s interval served as the baseline, and the 0 to 20 s interval as the analysis window. Spectral power was computed using a short-time Fourier transform (STFT) with a fixed-length Hanning window, providing consistent temporal resolution across frequencies. ERD/ERS values were calculated as log-transformed changes in spectral power (in decibels) relative to the pre-stimulus baseline. The calculated ERD/ERS values were averaged across six frequency bands (delta: 0.5–4 Hz, theta: 4–8 Hz, alpha: 8–12 Hz, beta: 12–30 Hz, gamma: 30–55 Hz, and all frequency: 0.5–55 Hz). The ERD/ERS values for the six frequency bands were averaged over time for quantification analysis. To avoid pseudo-replication, the ERSP values were averaged across the five repetitions for each formulation, resulting in one representative value per condition per participant used for subsequent analysis. For each of the three positive formulations (P1, P2, P3), ERD/ERS values and preference scores were corrected by subtracting those of the negative formulation (N).

### Correlation analysis

Pearson correlation analysis was used when there was only one independent variable and one dependent variable to assess the relationship and statistical significance between two measures. When two or more independent variables were involved, multiple correlation analysis was conducted. For multiple correlation analysis, the weights that best linearize the relationship between multiple independent variables and the dependent variable were calculated thorough multiple regression analysis. The predicted values of the dependent variable were then obtained by multiplying the independent variables by their corresponding weights. Subsequently, Pearson correlation analysis between the predicted and actual dependent variable was then used to assess statistical significance. To reduce the risk of false positives due to multiple correlation tests, permutation-based statistical testing (1,000 permutations) was performed across each frequency bands and electrodes to obtain adjusted *p*-values.

**Figure 3:**
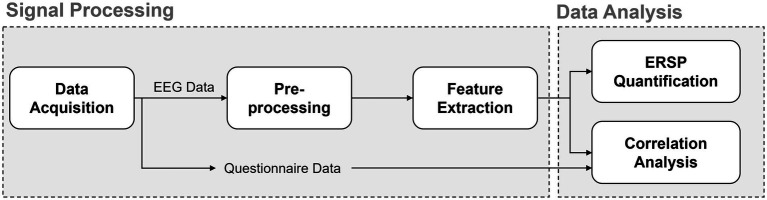
Overall workflow of signal processing and data analysis. EEG and questionnaire data were acquired during the experiment. EEG data were preprocessed and used for feature extraction, followed by ERSP quantification and correlation analysis to examine the relationship between brain activity and subjective evaluation.

In this study, Pearson correlation analysis was first used to examine the relationship between ERD/ERS values (i.e., power values for each frequency band and electrode) and the questionnaire-based preference scores. Frequency bands that exhibited significant results were then combined in a multiple correlation analysis to further investigate the relationship with preference scores. The overall process from data collection to analysis is summarized in Figure 3.

### Statistical analysis

Statistical analysis was performed to compare the questionnaire-based preference scores for the four different formulations (P1, P2, P3, N). A Friedman test was used for group comparisons, followed by Wilcoxon signed-rank tests to evaluate pairwise differences. Post-hoc *p*-values were corrected using the Bonferroni method. All statistical analyses were conducted in MATLAB R2017b (MathWorks, Natick, MA, USA).

## Results

**Figure 4:**
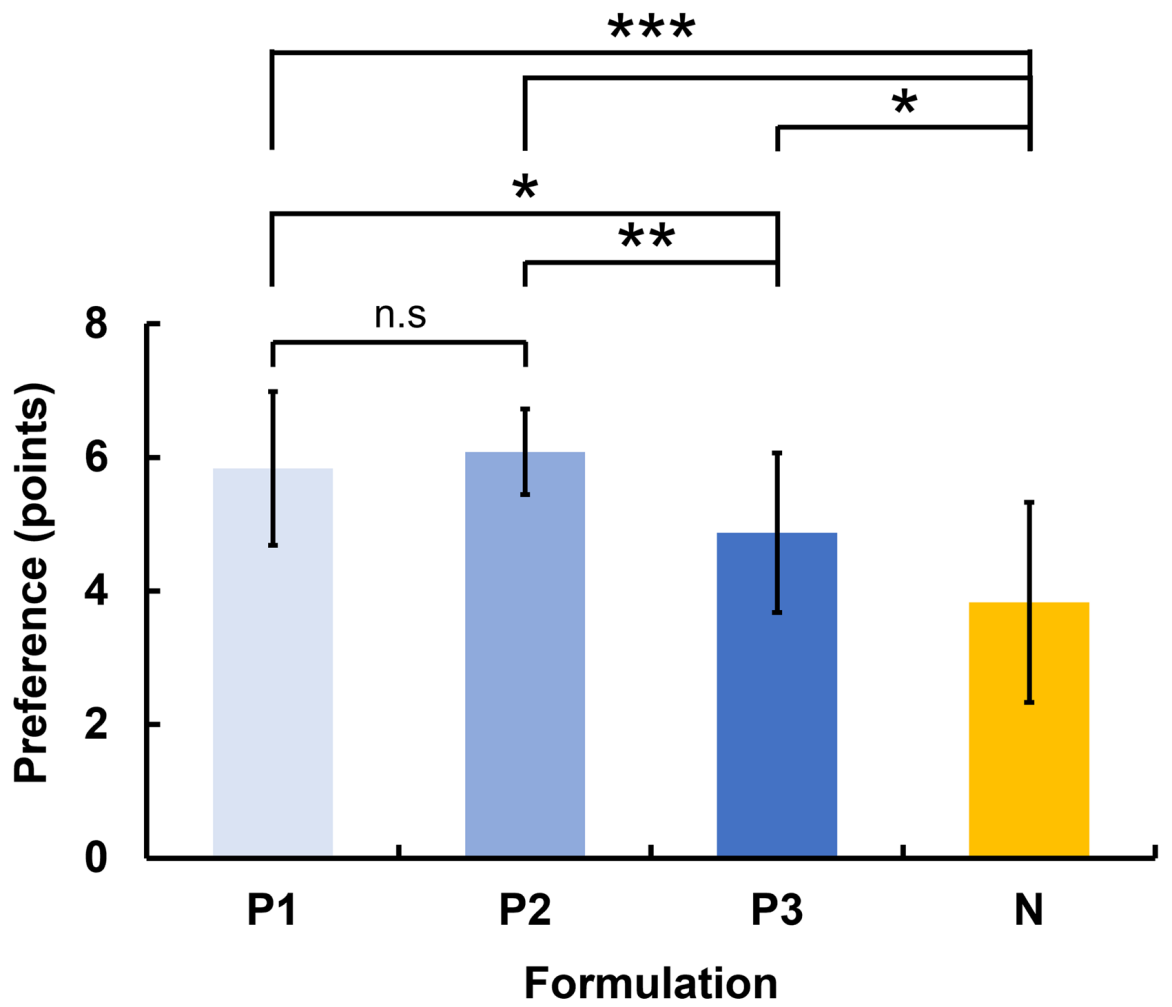
Mean and standard deviations of preference scores for each formulation based on a 7-point scale (*p < 0.05, **p < 0.01, ***p < 0.001). The negative (N) formulation showed significantly lower scores than all positive (P1, P2, P3) formulations. Among the positive formulations, P1 and P2 formulations showed significantly higher scores than P3 formulation. n.s indicates not significant (*p* > 0.05).

Figure 4 shows the preference scores for the four different formulations on the 7-point scale. The negative (N) formulation had significantly lower preference scores than the positive (P1, P2) formulations, and no significant difference was observed between the P1 formulation and P2 formulation (Friedman test: χ^2^(3, *n* = 116) = 52.32, Bonferroni corrected *p* < 0.05). In contrast, P3 formulation, which was originally expected to be a positive formulation based on its characteristics, showed a significant preference difference compared to P1 and P2, yet no significant difference from the negative formulation (N). This finding indicates that P3 was more closely aligned with the negative formulation in terms of subjective preference. Therefore, in subsequent analyses, positive formulations were divided both by their intended characteristics (P1 + P2 + P3) and by their actual questionnaire results (P1 + P2).

**Figure 5:**
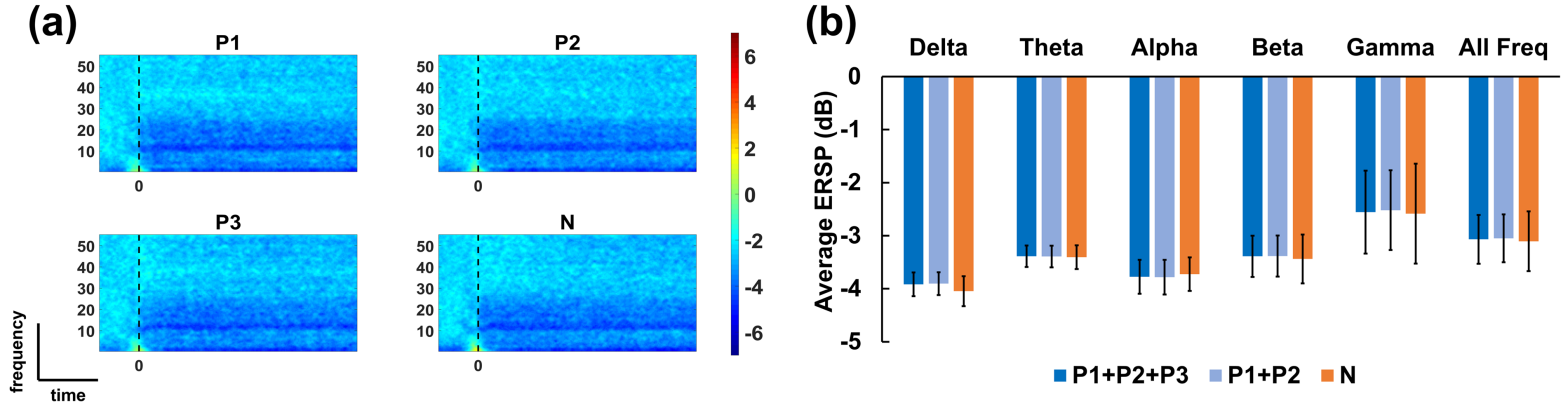
**(a)** Grand-average ERSP maps of the four formulations and **(b)** mean ERSP values across all channels for six frequency bands. The negative values observed across all frequency bands indicate prominent ERD activity. Across all conditions, no statistically meaningful differences were found.

Figure 5 shows the grand-average ERSP maps alongside the averaged quantitative ERSP values across all channels by frequency for both positive and negative formulations. The positive formulations were separated into two groups: those categorized by formulation characteristics (P1 + P2 + P3) and those categorized based on questionnaire results (P1 + P2). ERD/ERS values were consistently negative across all frequency bands and experimental conditions, indicating a predominant ERD pattern related to tactile sensation (Chen et al., 2019; Yakovlev et al., 2023; Morozova et al., 2024). No significant differences were observed among the conditions in terms of brain activity itself.

**Figure 6:**
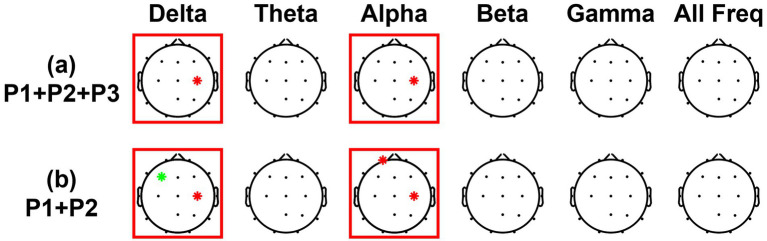
Correlation analysis results for **(a)** P1 + P2 + P3 and **(b)** P1 + P2 across different channels and frequency bands. Red asterisks indicate positive correlations, and the green asterisk indicates a negative correlation. Both cases showed significantly positive correlations in the delta and alpha bands at C4.

Figure 6 represents the results of Pearson correlation analysis between preference scores and ERD values. As previously mentioned, the ERD values and preference scores for each positive formulation (P1, P2, P3) were corrected by subtracting those of the negative formulation (N). Correlations were evaluated for each frequency band and channel, and significant findings are illustrated in topographic maps. Red asterisks indicate significant positive correlations, while green asterisks indicate significant negative correlations. In both analyses—using P1 + P2 + P3 and P1 + P2—significant correlations emerged in the delta and alpha bands at the C4 electrode located in the right sensorimotor region. Moreover, when the analysis was restricted to only P1 and P2, additional significant correlations were found at F3 in the delta band and at Fp1 in the alpha band.

**Figure 7:**
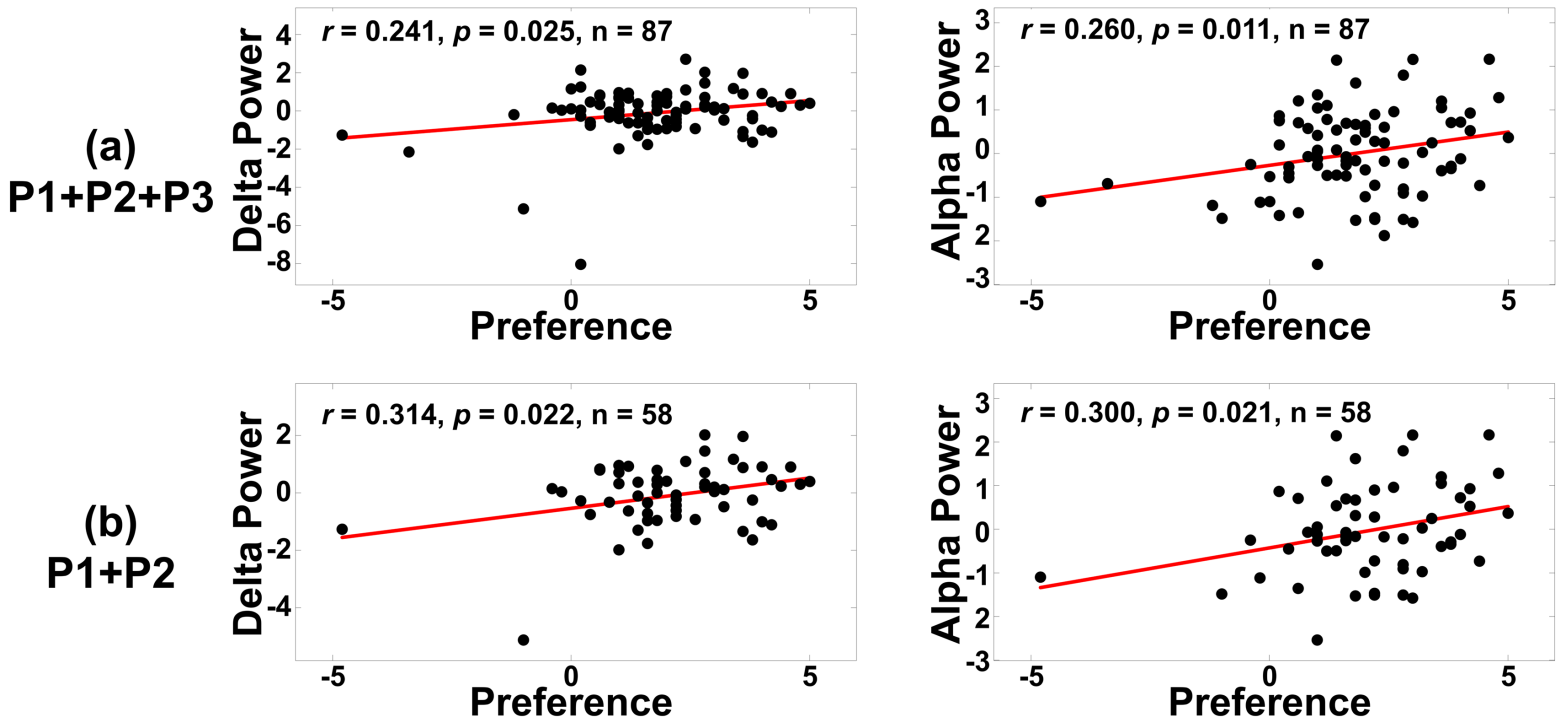
Correlation analysis results for delta and alpha bands at C4, comparing two cases: **(a)** characteristic-based positive formulations (P1 + P2 + P3) and **(b)** questionnaire-based positive formulations (P1 + P2). The questionnaire-based formulation (P1 + P2) showed stronger correlations compared to the characteristic-based formulation (P1 + P2 + P3).

Figure 7 provides the detailed correlation results focusing on the delta and alpha bands at C4, where consistent significance was observed. For the case using characteristic-based positive formulations (P1 + P2 + P3), the Pearson’s correlation coefficients (*r*) for the delta and alpha bands at C4 were 0.241 and 0.260, respectively (delta: *r* = 0.241, *p* = 0.024; alpha: *r* = 0.260, *p* = 0.015). On the other hand, when questionnaire-based positive formulation (P1 + P2) were analyzed, the correlation coefficients for delta and alpha bands increased to 0.314 and 0.300, respectively (delta: *r* = 0.314, *p* = 0.016; alpha: *r* = 0.300, *p* = 0.022), indicating stronger correlations compared to the analysis with characteristic-based positive formulations.

**Figure 8:**
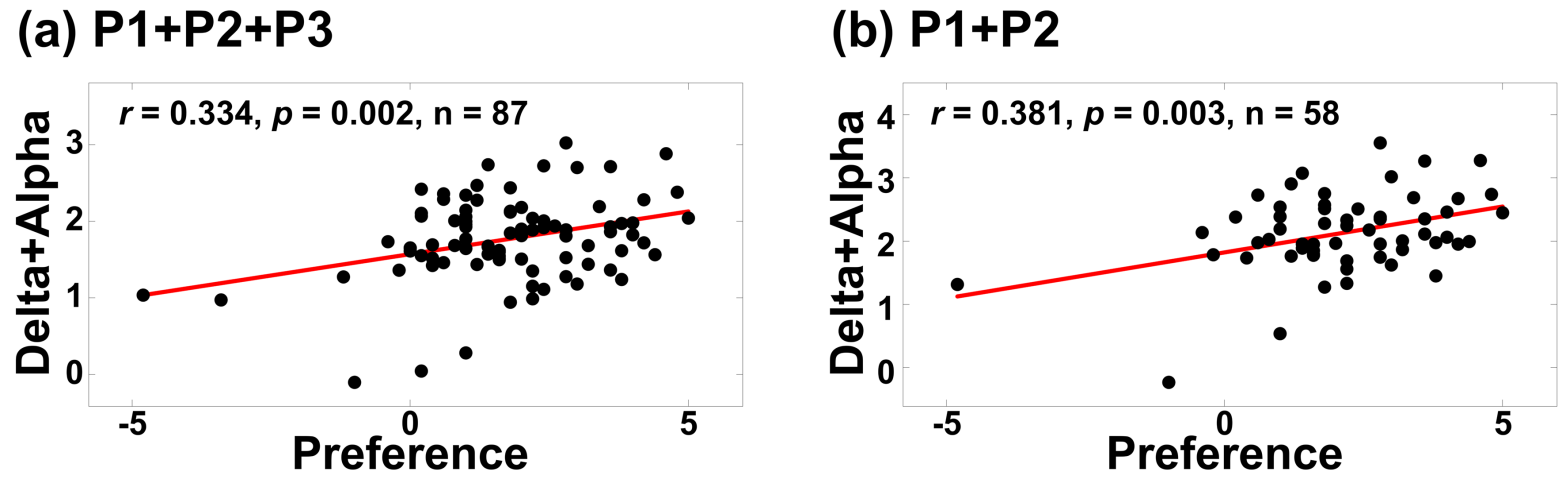
Multiple correlation analysis results at C4, incorporating both delta and alpha ERD values: **(a)** P1 + P2 + P3, **(b)** P1 + P2. Stronger correlations were observed for P1 + P2 and overall, the multiple correlation analysis outperformed the frequency-specific approaches.

Figure 8 shows the results of multiple correlation analysis for the C4 electrode, combining both delta and alpha power values (which were individually significant in the frequency-specific analyses). Similar to previous comparisons, two scenarios were considered: (1) P1 + P2 + P3 and (2) P1 + P2. The analysis revealed that using only P1 and P2 formulations again produced stronger correlations than using all three positive formulations (P1 + P2 + P3: *r* = 0.334, *p* = 0.002; P1 + P2: *r* = 0.381, *p* = 0.003). Notably, these multiple correlation coefficients exceeded those obtained from the frequency-specific analyses.

## Discussion

In this study, we investigated how different textures induced by different cosmetic formulations influence brain activity as measured by EEG and how these neural responses correlate with subjective preferences. Consistent with our initial expectations, participants rated the negative formulation significantly lower than the positive ones (except for P3 formulation), and the normalized EEG data consistently showed statistically significant correlations with preference in the delta and alpha bands at the C4 electrode.

The C4 electrode is located over the somatosensory cortex, which plays a key role in processing tactile information such as touch and pressure, and has also been associated with affective modulation (Pihko et al., 2004; Kropf et al., 2018; Yao et al., 2019; Eldeeb et al., 2020). In this study, participants applied the products on their left arm, thereby activating the contralateral (right) somatosensory area (Mashrur et al., 2022; Zhang et al., 2025). Notably, brain activity in this contralateral somatosensory area showed a significant correlation with subjective preferences. Given that the texture sensation induced by cosmetic application influences subjective product evaluations, the correlations detected at the C4 electrode are likely attributable to sensory-processing mechanisms in this region, potentially influenced by emotional components. In addition, some previous studies have suggested that activity in the C4 region may be involved in distinguishing emotional states such as positive and negative valence (Jiang et al., 2022; Tao et al., 2023), indicating a possible link between this region and emotional processing in tactile experience.

However, since emotional states are inherently abstract—and especially because studies directly linking tactile sensations induced by cosmetic formulations to emotion-related EEG responses remain limited—we acknowledge that this interpretation is still indirect and should be approached with caution. Specially, it cannot be entirely ruled out that motor-related signals from right-hand movement during self-application contributed to the EEG responses, as such movements can elicit bilateral cortical activation (Yuan et al., 2010; Hasegawa et al., 2017; Bundy and Leuthardt, 2019). Nonetheless, since motor activity of the dominant right hand is generally associated with stronger activation in the contralateral (left) hemisphere (van den Berg et al., 2011; Li et al., 2015; Ramos-Murguialday and Birbaumer, 2015), the right-hemisphere responses observed in this study are more likely driven by somatosensory rather than motor processes. Nevertheless, some overlap between motor- and somatosensory-related activity is inevitable in our design, although its potential influence on the present findings is likely minimal.

Significant correlations were found in the delta and alpha frequency bands. Alpha oscillations in sensorimotor regions generally exhibit ERD in the hemisphere contralateral to the point of stimulation (McFarland and Wolpaw, 2011; Chen et al., 2019; Hakim et al., 2021; Raiesdana and Mousakhani, 2022; Fabio et al., 2024), including in the somatosensory cortex corresponding to a specific skin area (Cheyne et al., 2003; Neuper et al., 2006; Haegens et al., 2014; Schubert et al., 2015). Therefore, in this study, the cosmetic formulation applied to the left arm can be seen as inducing sensory responses in the alpha power in the contralateral hemisphere, the right hemisphere. Furthermore, recent research has shown that delta activity can be relevant during tactile discrimination tasks (Abderrahmane et al., 2020; Taleei et al., 2023). In line with these findings, we observed significant delta-band correlations with textures that were relatively smooth, suggesting that delta-band features may be sensitive to subtle tactile variations.

When we restricted our analysis to only the two most preferred formulations (P1 and P2) versus the negative formulation, which were the positive formulation selected through the questionnaire, we found stronger correlations between EEG data and preference scores. The results highlight the discrepancy between the positive formulation criterion based on formulation characteristics and subjective questionnaires. Despite the similarity in texture and external characteristic of the cosmetic formulations, the preferences were not necessarily similar, suggesting that evaluating preferences based solely on the external texture of cosmetic formulations is challenging. To further clarify why P3 showed lower preference scores despite its physical similarity to P1 and P2, we analyzed the sensory questionnaire data collected alongside the preference ratings. The questionnaire assessed five attributes: spreadability, moisture, smoothness, oiliness, and stickiness. While P1 and P2 exhibited comparable scores across all five sensory items, P3 significantly differed from at least one of them on each attribute. Notably, when compared with the negative formulation (N), P3 did not differ significantly in three of five items—spreadability, moisture, and oiliness—suggesting that participants may have perceived P3 as more similar to N in terms of sensory experience. Since sensory impressions can significantly influence subjective preferences, these findings help explain why P3 received lower preference scores than P1 and P2, despite similar physical characteristics. The detailed statistical analyses related to the sensory questionnaire are provided in the Supplementary materials (Supplementary Figure S1).

Moreover, multiple correlation analysis using both delta and alpha power increased the predictive strength for subjective preferences relative to frequency-specific analyses alone. These results underscore the potential of combining multiple EEG features to enhance the prediction of consumer choices related to cosmetic formulations. Additionally, significant correlations were observed in the left frontal region for both delta and alpha frequency bands. The left frontal region is known to be associated with emotional processing, where an increase in alpha power and a decrease in delta power are observed during positive emotional states, whereas the opposite pattern occurs during negative emotional states (Burgdorf and Moskal, 2023; Chen et al., 2023). The findings of this study are consistent with previous research, and the fact that significant correlations were observed only in the P1 + P2 condition suggests that this trend becomes more evident as positive preference increases.

To further explore the temporal dynamics of EEG responses, we segmented the stimulation period into early (0–10 s) and late (10–20 s) phases. Although some preference-related correlations appeared in both phases, their spatial distributions differed. These differences may reflect the temporal evolution of tactile and affective processing. The early phase likely involves rapid encoding of primary somatosensory impressions—such as smoothness, and stickiness—processed in frontal and parietal regions associated with attention (Pleger et al., 2006; Adams et al., 2019). In contrast, the late phase may reflect higher-order integration processes involving prior experience and subjective evaluation (Ku et al., 2007; Wei et al., 2024), thereby engaging more sensorimotor-related regions such as C4. In fact, the late phase revealed consistent and robust correlations at the C4 channel, aligning well with the results from the full 20-s window. In contrast, theta-band correlations emerged in non-overlapping channels between early and late phases, suggesting that theta oscillations may be involved in distinct cognitive mechanisms at each time point. The time-segmented analysis results are presented in the Supplementary materials (Supplementary Figure S2, Supplementary Figure T1). Collectively, these findings underscore the potential of time-resolved EEG analysis in advancing affective neuroscience and preference prediction research.

This study also highlighted the feasibility of using dry EEG systems in a texture research context. Although dry electrodes eliminate the need for conductive gels, reducing setup time and improving long-term stability, they are still less common in applied research, especially for experiments involving touch (Lin et al., 2008; Liao et al., 2012; Lopez-Gordo et al., 2014; Faadhilah Afif et al., 2020). Our findings suggest that dry EEG equipment can provide reliable data, thereby supporting the broader application of EEG in everyday scenarios.

There are several limitations to our study. First, the experiment was conducted on female participants, who generally report higher engagement with cosmetic products and higher sensitivity to tactile texture than men. While this sampling approach helped reduce variability in subjective responses, it may limit the generalizability of the findings to male populations. Future studies should include male participants to determine whether the observed EEG–preference relationships are consistent across genders. Second, participants were free to use varying application methods (e.g., rubbing or tapping), which might have introduced variability in tactile stimulation (Greco et al., 2019; Eldeeb et al., 2020). This decision was made to enhance the ecological validity of the study by reflecting real-life usage scenarios in which cosmetic products are typically applied without standardized force or speed. Tactile sensations during application arise not only from the formulation itself, but also from its interaction with user-specific application styles. Allowing participants to apply the formulations naturally enabled a more authentic evaluation of the sensorial characteristics which in turn influence participants’ subjective preferences and neural responses. While such behavioral variation could serve as a potential confound, we minimized its impact by averaging EEG responses across repeated trials and observed consistent application patterns within individuals. Future studies may benefit from directly comparing standardized and naturalistic application conditions to better distinguish the effects of behavioral factors and formulation-driven responses.

Overall, these findings demonstrate that EEG, particularly measurements taken from the somatosensory cortex in the delta and alpha bands, can capture meaningful individual preferences for different cosmetic formulations. This suggests that neurophysiological signals may serve as an objective and real-time supplement to traditional consumer evaluations in cosmetics research.

## Conclusion

In this study, we investigated the correlation between EEG data and subjective preference ratings for cosmetic formulations with different textures. Our research findings identified significant relationships in the somatosensory cortex associated with tactile stimuli and in the delta and alpha bands, underscoring the importance of texture sensation in consumer choice. To the best of our knowledge, this is the first study to provide a clear neurophysiological interpretation by correlating EEG-based brain responses specifically to cosmetic textures with subjective preferences. Furthermore, by focusing exclusively on texture sensation and excluding other sensory elements such as scent and color, our approach helps identify brain activity patterns related to texture-driven product preference. These findings can enhance our understanding of different consumer groups and aid in the design of cosmetic products with textures that evoke stronger emotional satisfaction. In addition, these findings can help bridge the gap between neuroscience and marketing by providing a valuable tool to capture texture-specific consumer preferences that traditional surveys or interviews may overlook. In future studies, we plan to develop a more comprehensive system that classifies and evaluates preferences for cosmetic formulations using a broader range of EEG-driven features. By integrating these neurophysiological measures with traditional consumer research, we may pave the way for highly individualized cosmetic product recommendations and improved design processes in the cosmetics industry.

## Data Availability

The raw data supporting the conclusions of this article will be made available by the authors, without undue reservation.

## References

[ref1] Abderrahmane Z. Ganesh G. Crosnier A. Cherubini A. (2020). A deep learning framework for tactile recognition of known as well as novel objects. IEEE Trans. Ind. Inform. 16, 423–432. doi: 10.1109/TII.2019.2898264

[ref2] Adams M. S. Andrew D. Staines W. R. (2019). The contribution of the prefrontal cortex to relevancy-based gating of visual and tactile stimuli. Exp. Brain Res. 237, 2747–2759. doi: 10.1007/s00221-019-05633-9, PMID: 31435693

[ref3] Alpine berry watery oil-free gel cream (2025). Available online at: https://www.aritaum.com/shop/pr/productView/P0000000000000041026.do (Accessed March 19, 2025).

[ref4] Aquaphor® baby healing ointment (14 oz.) (2025). Available online at: https://www.aquaphorus.com/products/aquaphor/aquaphor-baby-healing-ointment-14oz (Accessed March 19, 2025).

[ref5] Ares G. Mawad F. Giménez A. Maiche A. (2014). Influence of rational and intuitive thinking styles on food choice: preliminary evidence from an eye-tracking study with yogurt labels. Food Qual. Prefer. 31, 28–37. doi: 10.1016/j.foodqual.2013.07.005

[ref6] Bos D. O. (2006). EEG-based emotion recognition. Influ. Vis. Audit. Stimuli 56, 1–17. Available online at: https://www.researchgate.net/publication/237777779_EEG-based_Emotion_Recognition

[ref7] Bradley C. Joyce N. Garcia-Larrea L. (2016). Adaptation in human somatosensory cortex as a model of sensory memory construction: a study using high-density EEG. Brain Struct. Funct. 221, 421–431. doi: 10.1007/s00429-014-0915-5, PMID: 25352155

[ref8] Bundy D. T. Leuthardt E. C. (2019). The cortical physiology of ipsilateral limb movements. Trends Neurosci. 42, 825–839. doi: 10.1016/j.tins.2019.08.008, PMID: 31514976 PMC6825896

[ref9] Burgdorf J. S. Moskal J. R. (2023). A prefrontal cortex alpha/delta switch controls the transition from positive to negative affective states. Discov. Ment. Health 3:19. doi: 10.1007/s44192-023-00044-3, PMID: 37861869 PMC10564693

[ref10] Cavill S. Bryden P. (2003). Development of handedness: comparison of questionnaire and performance-based measures of preference. Brain Cogn. 53, 149–151. doi: 10.1016/S0278-2626(03)00098-8, PMID: 14607136

[ref11] Chen M. L. Fu D. Boger J. Jiang N. (2019). Age-related changes in vibro-tactile EEG response and its implications in BCI applications: a comparison between older and younger populations. IEEE Trans. Neural Syst. Rehabil. Eng. 27, 603–610. doi: 10.1109/TNSRE.2019.2890968, PMID: 30872232

[ref12] Chen J. Wang X. Huang C. Hu X. Shen X. Zhang D. (2023). A large finer-grained affective computing EEG dataset. Sci Data 10:740. doi: 10.1038/s41597-023-02650-w, PMID: 37880266 PMC10600242

[ref13] Cheyne D. Gaetz W. Garnero L. Lachaux J.-P. Ducorps A. Schwartz D.. (2003). Neuromagnetic imaging of cortical oscillations accompanying tactile stimulation. Cogn. Brain Res. 17, 599–611. doi: 10.1016/S0926-6410(03)00173-3, PMID: 14561448

[ref14] Churchill A. Behan J. (2010). Comparison of methods used to study consumer emotions associated with fragrance. Food Qual. Prefer. 21, 1108–1113. doi: 10.1016/j.foodqual.2010.07.006

[ref15] Color correcting technology (2025). Available online at: https://prd-en-int.apgroup.com/int/en/about-us/research-innovation/rni/beauty-research-innovation/beauty-research-innovation-13.html (Accessed March 13, 2025).

[ref16] Cuesta F. Paida G. Buele I. (2020). Influence of olfactory and visual sensory stimuli in the perfume-purchase decision. Int. Rev. Manag. Mark. 10, 63–71. doi: 10.32479/irmm.8963

[ref17] Diwoux A. Gabriel D. Bardel M.-H. Ben Khalifa Y. Billot P.-É. (2024). Neurophysiological approaches to exploring emotional responses to cosmetics: a systematic review of the literature. Front. Hum. Neurosci. 18:1443001. doi: 10.3389/fnhum.2024.1443001, PMID: 39502789 PMC11534817

[ref18] Dr.JCOS INC. (2025). Available online at: http://www.drjcos.com (Accessed March 14, 2025).

[ref19] Dry EEG headset (2025). CGX. Available online at: https://www.cgxsystems.com/quick-20r-v2 (Accessed March 14, 2025).

[ref20] Eldeeb S. Weber D. Ting J. Demir A. Erdogmus D. Akcakaya M. (2020). EEG-based trial-by-trial texture classification during active touch. Sci. Rep. 10:20755. doi: 10.1038/s41598-020-77439-7, PMID: 33247177 PMC7699648

[ref21] Faadhilah Afif N. Harke Pratama S. Haryanto F. Nurul Khotimah S. (2020). Comparison of wet and dry EEG electrodes based on brain signals characterization in temporal and anterior frontal areas using audio stimulation. J. Phys. Conf. Ser. 1505:012069. doi: 10.1088/1742-6596/1505/1/012069

[ref22] Fabio C. Salemme R. Farnè A. Miller L. E. (2024). Alpha oscillations reflect similar mapping mechanisms for localizing touch on hands and tools. iScience 27:109092. doi: 10.1016/j.isci.2024.109092, PMID: 38405611 PMC10884914

[ref23] Field T. Diego M. Hernandez-Reif M. Cisneros W. Feijo L. Vera Y.. (2005). Lavender fragrance cleansing gel effects on relaxation. Int. J. Neurosci. 115, 207–222. doi: 10.1080/00207450590519175, PMID: 15764002

[ref24] Frey J. H. Fontana A. (1991). The group interview in social research. Soc. Sci. J. 28, 175–187. doi: 10.1016/0362-3319(91)90003-M

[ref25] Gabriel D. Merat E. Jeudy A. Cambos S. Chabin T. Giustiniani J.. (2021). Emotional effects induced by the application of a cosmetic product: a real-time electrophysiological evaluation. Appl. Sci. 11:4766. doi: 10.3390/app11114766

[ref26] Golnar-Nik P. Farashi S. Safari M.-S. (2019). The application of EEG power for the prediction and interpretation of consumer decision-making: a neuromarketing study. Physiol. Behav. 207, 90–98. doi: 10.1016/j.physbeh.2019.04.025, PMID: 31047949

[ref27] Greco A. Guidi A. Bianchi M. Lanata A. Valenza G. Scilingo E. P. (2019). Brain dynamics induced by pleasant/unpleasant tactile stimuli conveyed by different fabrics. IEEE J. Biomed. Health Inform. 23, 2417–2427. doi: 10.1109/JBHI.2019.2893324, PMID: 30668509

[ref28] Haegens S. Vázquez Y. Zainos A. Alvarez M. Jensen O. Romo R. (2014). Thalamocortical rhythms during a vibrotactile detection task. Proc. Natl. Acad. Sci. USA 111, E1797–E1805. doi: 10.1073/pnas.1405516111, PMID: 24733899 PMC4035942

[ref29] Hakim A. Klorfeld S. Sela T. Friedman D. Shabat-Simon M. Levy D. J. (2021). Machines learn neuromarketing: improving preference prediction from self-reports using multiple EEG measures and machine learning. Int. J. Res. Mark. 38, 770–791. doi: 10.1016/j.ijresmar.2020.10.005

[ref30] Hasegawa K. Kasuga S. Takasaki K. Mizuno K. Liu M. Ushiba J. (2017). Ipsilateral EEG mu rhythm reflects the excitability of uncrossed pathways projecting to shoulder muscles. J. Neuroeng. Rehabil. 14:85. doi: 10.1186/s12984-017-0294-2, PMID: 28841920 PMC5574148

[ref31] Jenke R. Peer A. Buss M. (2014). Feature extraction and selection for emotion recognition from EEG. IEEE Trans. Affect. Comput. 5, 327–339. doi: 10.1109/TAFFC.2014.2339834

[ref32] Jiang L. Siriaraya P. Choi D. Zeng F. Kuwahara N. (2022). Electroencephalogram signals emotion recognition based on convolutional neural network-recurrent neural network framework with channel-temporal attention mechanism for older adults. Front. Aging Neurosci. 14:945024. doi: 10.3389/fnagi.2022.945024, PMID: 36212045 PMC9535340

[ref33] Khondakar M. F. K. Sarowar M. H. Chowdhury M. H. Majumder S. Hossain M. A. Dewan M. A. A.. (2024). A systematic review on EEG-based neuromarketing: recent trends and analyzing techniques. Brain Inform. 11:17. doi: 10.1186/s40708-024-00229-8, PMID: 38837089 PMC11153447

[ref34] Khurana V. Gahalawat M. Kumar P. Roy P. P. Dogra D. P. Scheme E.. (2021). A survey on neuromarketing using EEG signals. IEEE Trans. Cogn. Dev. Syst. 13, 732–749. doi: 10.1109/TCDS.2021.3065200

[ref35] Kropf E. Syan S. K. Minuzzi L. Frey B. N. (2018). From anatomy to function: the role of the somatosensory cortex in emotional regulation. Braz. J. Psychiatr. 41, 261–269. doi: 10.1590/1516-4446-2018-0183 PMC6794131 30540029

[ref36] Ku Y. Ohara S. Wang L. Lenz F. A. Hsiao S. S. Bodner M.. (2007). Prefrontal cortex and somatosensory cortex in tactile crossmodal association: an independent component analysis of ERP recordings. PLoS One 2:e771. doi: 10.1371/journal.pone.0000771, PMID: 17712419 PMC1942117

[ref37] Li L. Wang J. Xu G. Li M. Xie J. (2015). The study of object-oriented motor imagery based on EEG suppression. PLoS One 10:e0144256. doi: 10.1371/journal.pone.0144256, PMID: 26641241 PMC4671551

[ref38] Liao L.-D. Chen C.-Y. Wang I.-J. Chen S.-F. Li S.-Y. Chen B.-W.. (2012). Gaming control using a wearable and wireless EEG-based brain-computer interface device with novel dry foam-based sensors. J. Neuroeng. Rehabil. 9:5. doi: 10.1186/1743-0003-9-5, PMID: 22284235 PMC3283495

[ref39] Lin C.-T. Ko L.-W. Chiou J.-C. Duann J.-R. Huang R.-S. Liang S.-F.. (2008). Noninvasive neural prostheses using mobile and wireless EEG. Proc. IEEE 96, 1167–1183. doi: 10.1109/JPROC.2008.922561

[ref40] Lopez-Gordo M. A. Sanchez-Morillo D. Valle F. P. (2014). Dry EEG electrodes. Sensors 14, 12847–12870. doi: 10.3390/s140712847, PMID: 25046013 PMC4168519

[ref41] Mashrur F. R. Rahman K. M. Miya M. T. I. Vaidyanathan R. Anwar S. F. Sarker F.. (2022). BCI-based consumers’ choice prediction from EEG signals: an intelligent neuromarketing framework. Front. Hum. Neurosci. 16:861270. doi: 10.3389/fnhum.2022.861270, PMID: 35693537 PMC9177951

[ref42] McFarland D. J. Wolpaw J. R. (2011). Brain-computer interfaces for communication and control. Commun. ACM 54, 60–66. doi: 10.1145/1941487.1941506, PMID: 21984822 PMC3188401

[ref43] Morozova M. Yakovlev L. Syrov N. Lebedev M. Kaplan A. (2024). Tactile imagery affects cortical responses to vibrotactile stimulation of the fingertip. Heliyon 10:e40807. doi: 10.1016/j.heliyon.2024.e40807, PMID: 39698084 PMC11652922

[ref44] Neuper C. Wörtz M. Pfurtscheller G. (2006). ERD/ERS patterns reflecting sensorimotor activation and deactivation, Prog. Brain Res. 159, 211–222. doi: 10.1016/S0079-6123(06)59014-4 17071233

[ref45] Ouzir M. Chakir Lamrani H. Bradley R. L. El Moudden I. (2024). Neuromarketing and decision-making: classification of consumer preferences based on changes analysis in the EEG signal of brain regions. Biomed. Signal Process. Control. 87:105469. doi: 10.1016/j.bspc.2023.105469

[ref46] Phan K. L. Wager T. Taylor S. F. Liberzon I. (2002). Functional neuroanatomy of emotion: a meta-analysis of emotion activation studies in PET and fMRI. NeuroImage 16, 331–348. doi: 10.1006/nimg.2002.1087, PMID: 12030820

[ref47] Pihko E. Lauronen L. Wikström H. Taulu S. Nurminen J. Kivitie-Kallio S.. (2004). Somatosensory evoked potentials and magnetic fields elicited by tactile stimulation of the hand during active and quiet sleep in newborns. Clin. Neurophysiol. 115, 448–455. doi: 10.1016/S1388-2457(03)00349-3, PMID: 14744587

[ref48] Pleger B. Ruff C. C. Blankenburg F. Bestmann S. Wiech K. Stephan K. E.. (2006). Neural coding of tactile decisions in the human prefrontal cortex. J. Neurosci. 26, 12596–12601. doi: 10.1523/JNEUROSCI.4275-06.2006, PMID: 17135421 PMC2636906

[ref49] PsychoPy (2025) PsychoPy. Available online at: https://www.psychopy.org/

[ref50] Pure artemisia calming water cream (2025). HANYUL. Available online at: https://www.hanyul.com/kr/ko/product/hanyul-pure-artemisia-calming-water-cream.html (Accessed March 19, 2025).

[ref51] Raiesdana S. Mousakhani M. (2022). An EEG-based neuromarketing approach for analyzing the preference of an electric car. Comput. Intell. Neurosci. 2022:9002101. doi: 10.1155/2022/9002101, PMID: 35341175 PMC8956417

[ref52] Ramos-Murguialday A. Birbaumer N. (2015). Brain oscillatory signatures of motor tasks. J. Neurophysiol. 113, 3663–3682. doi: 10.1152/jn.00467.2013, PMID: 25810484 PMC4468978

[ref53] Rideaux R. West R. K. Rangelov D. Mattingley J. B. (2023). Distinct early and late neural mechanisms regulate feature-specific sensory adaptation in the human visual system. Proc. Natl. Acad. Sci. USA 120:e2216192120. doi: 10.1073/pnas.2216192120, PMID: 36724257 PMC9963156

[ref54] Schubert J. T. W. Buchholz V. N. Föcker J. Engel A. K. Röder B. Heed T. (2015). Oscillatory activity reflects differential use of spatial reference frames by sighted and blind individuals in tactile attention. NeuroImage 117, 417–428. doi: 10.1016/j.neuroimage.2015.05.068, PMID: 26032885

[ref55] Subramanian S. De Moor K. Fiedler M. Koniuch K. Janowski L. (2023). Towards enhancing ecological validity in user studies: a systematic review of guidelines and implications for QoE research. Qual. User Exp. 8:6. doi: 10.1007/s41233-023-00059-2

[ref56] Tailored facial mask pack 3D printing system (2025). Available online at: https://prd-en-int.apgroup.com/int/en/about-us/research-innovation/rni/beauty-research-innovation/beauty-research-innovation-12.html (Accessed March 14, 2025).

[ref57] Taleei T. Nazem-Zadeh M.-R. Amiri M. Keliris G. A. (2023). EEG-based functional connectivity for tactile roughness discrimination. Cogn. Neurodyn. 17, 921–940. doi: 10.1007/s11571-022-09876-1, PMID: 37522039 PMC10374498

[ref58] Tao W. Li C. Song R. Cheng J. Liu Y. Wan F.. (2023). EEG-based emotion recognition via channel-wise attention and self attention. IEEE Trans. Affect. Comput. 14, 382–393. doi: 10.1109/TAFFC.2020.3025777

[ref59] van den Berg F. E. Swinnen S. P. Wenderoth N. (2011). Excitability of the motor cortex ipsilateral to the moving body side depends on spatio-temporal task complexity and hemispheric specialization. PLoS One 6:e17742. doi: 10.1371/journal.pone.0017742, PMID: 21408031 PMC3052419

[ref60] Wang F. Ma X. Cheng D. Gao L. Yao C. Lin W. (2024). Electroencephalography as an objective method for assessing subjective emotions during the application of cream. Skin Res. Technol. 30:e13692. doi: 10.1111/srt.13692, PMID: 38650354 PMC11035903

[ref61] Wei W. Benn R. A. Scholz R. Shevchenko V. Klatzmann U. Alberti F.. (2024). A function-based mapping of sensory integration along the cortical hierarchy. Commun. Biol. 7:1593. doi: 10.1038/s42003-024-07224-z, PMID: 39613829 PMC11607388

[ref62] Yadava M. Kumar P. Saini R. Roy P. P. Prosad Dogra D. (2017). Analysis of EEG signals and its application to neuromarketing. Multimed. Tools Appl. 76, 19087–19111. doi: 10.1007/s11042-017-4580-6

[ref63] Yakovlev L. Syrov N. Miroshnikov A. Lebedev M. Kaplan A. (2023). Event-related desynchronization induced by tactile imagery: an EEG study. ENeuro 10:ENEURO.0455-22.2023. doi: 10.1523/ENEURO.0455-22.2023, PMID: 37263791 PMC10275400

[ref64] Yao L. Sheng X. Mrachacz-Kersting N. Zhu X. Farina D. Jiang N. (2019). Sensory stimulation training for BCI system based on somatosensory attentional orientation. I.E.E.E. Trans. Biomed. Eng. 66, 640–646. doi: 10.1109/TBME.2018.2852755, PMID: 29993483

[ref65] Yuan H. Liu T. Szarkowski R. Rios C. Ashe J. He B. (2010). Negative covariation between task-related responses in alpha/beta-band activity and BOLD in human sensorimotor cortex: an EEG and fMRI study of motor imagery and movements. NeuroImage 49, 2596–2606. doi: 10.1016/j.neuroimage.2009.10.028, PMID: 19850134 PMC2818527

[ref66] Zhang S. Tang W. Fang X. Yang L. Zhang M. (2025). Tactile perception of rough surface based on skin friction and brain response. Tribol. Int. 202:110396. doi: 10.1016/j.triboint.2024.110396

