# Supplementary material for: Electroencephalography-based neural indicators of texture preference for cosmetic formulations
Source: Front Neurosci. 2025 Oct 29;19:1620806. doi: 10.3389/fnins.2025.1620806 (PMC12605486; doi:10.3389/fnins.2025.1620806)
Supplement: Supplementary file 1 [file Data_Sheet_1.PDF]

Supplementary Figure S1. Sensory evaluation scores of each formulation (P1, P2, P3, N) across five attributes: spreadability, moisture, smoothness, oiliness, and stickiness. P1 and P2 showed no significant differences across all items, whereas P3 differed significantly from either P1 or P2 on all five attributes. Notably, P3 and the negative formulation (N) did not differ significantly in spreadability, moisture, and oiliness, suggesting a perceptual similarity between them.

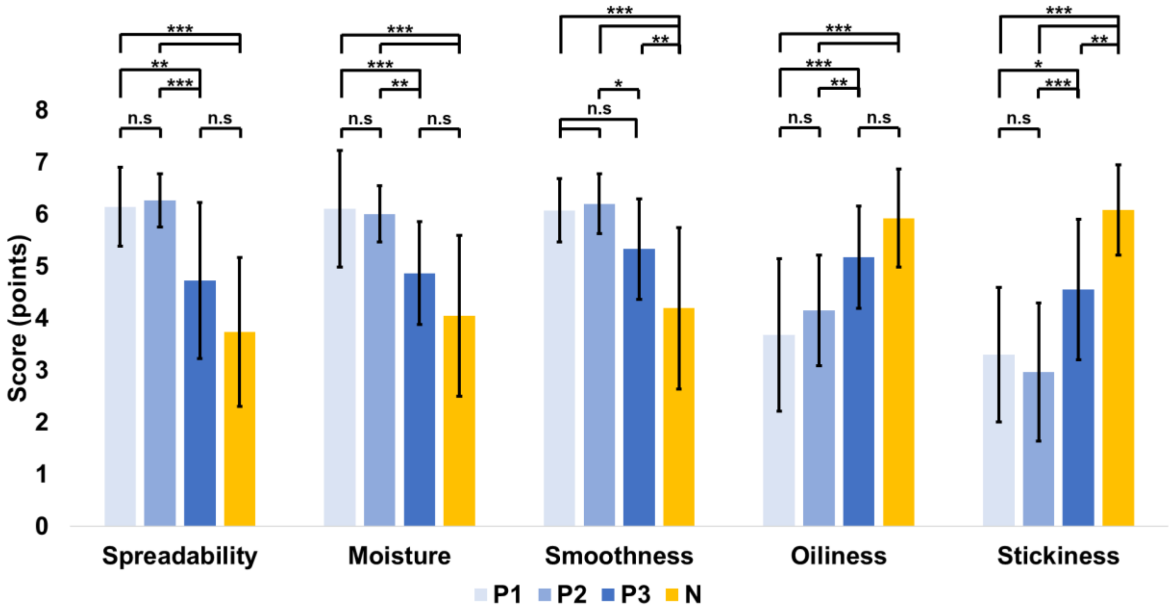

Supplementary Figure S2. Topographical maps of EEG–preference correlation coefficients during the early (0–10 s) and late (10–20 s) phases. Notable positive correlations in the alpha band at C4 appeared only in the late phase, while theta band correlations varied across channels between early and late phases.

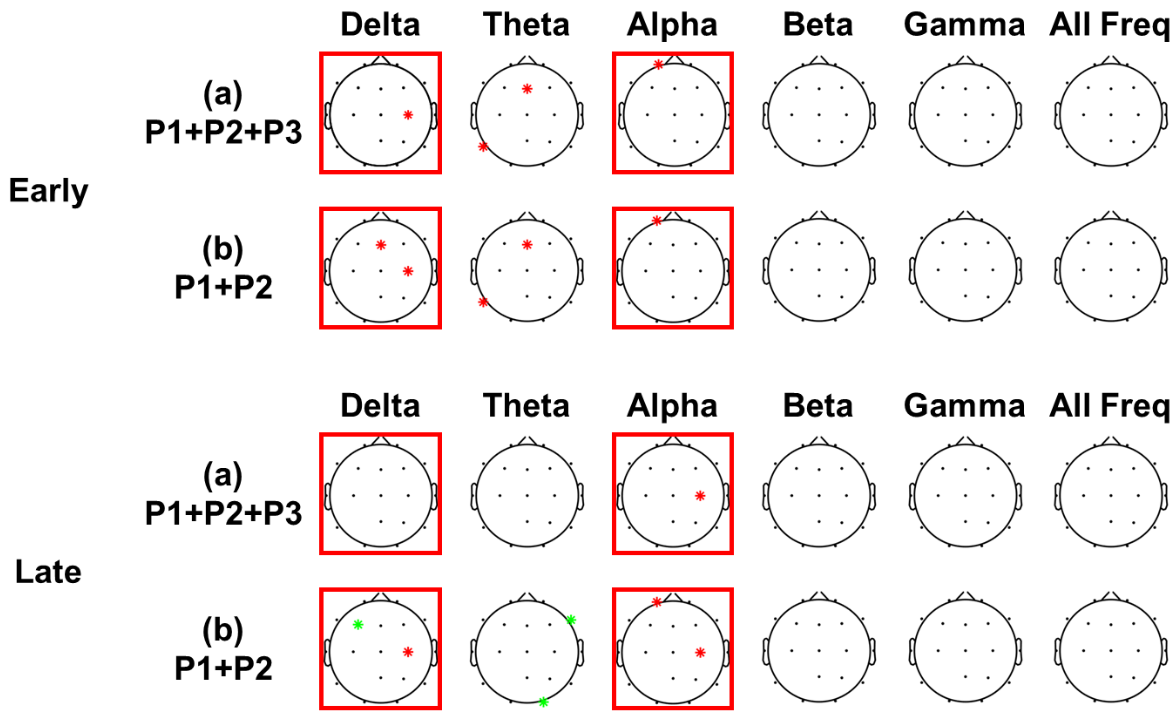

Supplementary Table S1. Summary of significant EEG-preference correlations across early and late time windows.

		**Delta**	**Theta**	**Alpha**
Early	P1+P2+P3	C4<br>(r = 0.252, *p* = 0.020)	Fz<br>(r = 0.255, *p* = 0.014)<br>P7<br>(r = 0.312, *p* = 0.007)	FP1<br>(r = 0.238, *p* = 0.024)
	P1+P2	Fz<br>(r = 0.274, *p* = 0.040)<br>C4<br>(r = 0.319, *p* = 0.016)	Fz<br>(r = 0.278, *p* = 0.034)<br>P7<br>(r = 0.319, *p* = 0.016)	FP1<br>(r = 0.396, *p* = 0.002)
Late	P1+P2+P3	-	-	C4<br>(r = 0.278, *p* = 0.012)
	P1+P2	F3<br>(r = -0.262, *p* = 0.049)<br>C4<br>(r = 0.270, *p* = 0.036)	F8<br>(r = -0.265, *p* = 0.046)<br>O2<br>(r = -0.264, *p* = 0.040)	FP1<br>(r = 0.279, *p* = 0.034)<br>C4<br>(r = 0.338, *p* = 0.004)
